# Restorative environments and humanized practice in oncology services

**DOI:** 10.47626/1679-4435-2025-1482

**Published:** 2025-10-01

**Authors:** Helder Matheus Alves Fernandes, Israel Coutinho Sampaio Lima, Lizandra Saraiva Borges, José Jackson Coelho Sampaio

**Affiliations:** 1 Centro de Ciências da Saúde, Universidade Estadual do Ceará, Fortaleza, CE, Brazil.; 2 Centro de Humanidades, Universidade Federal do Ceará, Fortaleza, CE, Brazil.

**Keywords:** occupational health, environmental psychology, oncology service, hospital, mental health, humanization of assistance, saúde ocupacional, psicologia ambiental, oncologia, saúde mental, humanização da assistência

## Abstract

This article critically examines how environmental psychology informs the design of restorative environments that foster humanized care among health professionals who treat people with cancer. It is a reflective, qualitative, exploratory study grounded in a critical analysis of the scientific literature. Articles, books, and theses on environmental psychology, work, restorative environments, and the humanization of oncology care were identified and selected using terms drawn from the Health Sciences Descriptors. The analysis was guided by Attention Restoration Theory and Stress Reduction Theory, which together frame the role of environmental psychology in physical and psychological well-being. The reflections indicate that, given the dynamic interplay between workers and their surroundings, the care environment (*ambiência*) plays a central role in promoting well-being and mental health among oncology professionals. Examining these interrelations shows how the care environment can mitigate stress, support mental health, and reduce physical and mental fatigue. For health professionals, appropriate environments are decisive for improving work quality and preventing emotional strain arising from protocol-driven, high-intensity routines. Further studies are warranted to deepen understanding of the relationships among workers, the care environment, and mental health, with the aim of advancing more effective and compassionate oncology care. In this context, environmental psychology provides a pertinent theoretical and practical framework for rethinking and improving the hospital care environment.

## Abstract (pt)

Este artigo tem por objetivo refletir criticamente sobre as contribuições da psicologia ambiental para a criação de ambientes restauradores que promovam o trabalho humanizado de profissionais de saúde na assistência a pessoas com câncer. Trata-se de um estudo de reflexão, de natureza qualitativa e exploratória, fundamentado em análise crítica da literatura científica. Artigos, livros e teses sobre psicologia ambiental, trabalho, ambientes restauradores e humanização do cuidado oncológico foram consultados e selecionados por meio de termos extraídos dos Descritores em Ciências da Saúde. A análise foi guiada pela Teoria da Restauração da Atenção e pela Teoria da Redução do Estresse, que embasam o papel da psicologia ambiental no bem-estar físico e psicológico. As reflexões indicam que, pela dinâmica de inter-relação entre trabalhador e ambiente, a ambiência exerce papel central na promoção do bem-estar e da saúde mental dos profissionais de oncologia. A leitura dessas inter-relações evidencia como a ambiência pode contribuir para mitigar o estresse, favorecer a saúde mental e reduzir a fadiga física e mental. Para profissionais da saúde, ambientes adequados tendem a ser determinantes na melhoria da qualidade do trabalho e na prevenção do desgaste emocional decorrente de rotinas intensas, frequentemente orientadas por protocolos. Recomenda-se a realização de novos estudos para aprofundar a compreensão da relação entre trabalhador, ambiente/ambiência e saúde mental, com vistas a um cuidado oncológico mais eficaz e compassivo. Nesse contexto, a psicologia ambiental oferece um arcabouço teórico e prático pertinente para repensar e aprimorar a ambiência hospitalar.

## INTRODUCTION

In the epistemological field of environmental psychology, the notion of restorative environments refers to spaces with specific — yet subjectively perceived — features that can promote users’ psychophysiological balance. Such environments foster attention restoration, help reduce stress/distress and physical and mental fatigue, and enhance the capacity for concentration, particularly in contexts marked by demanding, repetitive daily activities.^1^

Adopting an environmental psychology perspective as an organizational process is especially pertinent for health workers, who tend to operate according to protocol- and technique-driven standards in both professional practice and institutional settings.^2^

Research on restorative environments is grounded largely in two approaches that explain how natural or built settings can promote quality of life and well-being: Attention Restoration Theory (ART), developed by Rachel and Stephen Kaplan,^3^ and Stress Reduction Theory (SRT), proposed by Roger Ulrich.^4^ Incorporating these theoretical frameworks into environmental interventions supports psychological recovery and reduces cognitive overload, promoting mental rest and emotional regulation. In demanding work contexts, they thus constitute a relevant health-promotion strategy.

Health promotion is directly related to the effects of these environments on mental and emotional health, insofar as they provide spaces for relief, leisure, rest, comfort, and well-being, while ensuring adequate working conditions consistent with principles in health and medicine and their interfaces with society.^5^,^6^

Within oncology care, the relevance of restorative environments is even greater, given the rising incidence of cancer and the resulting demand for professionals. The International Agency for Research on Cancer reported global estimates of roughly 20 million new cases and 9.7 million deaths from cancer in 2022, as published in CA: A Cancer Journal for Clinicians.^7^ The number of survivors 5 years after diagnosis was estimated at 53.5 million, and approximately one in five people will develop cancer over a lifetime; about one in nine men and one in 12 women will die from the disease.^7^

In parallel, work absences due to mental disorders have increased substantially. Data from Brazil’s Ministry of Social Security show that, in 2023, disability benefits related to mental and behavioral disorders rose by 38% compared with 2022, reflecting the growth of psychological illness in the economically active population and its impact on productivity and public health.^8^

These repercussions intensify in oncology routines: workers are daily exposed to patients’ suffering, to confronting death, and to the high intensity of treatments. Added to this is the emotional burden associated with complex diagnoses and uncertain prognoses, which demands robust psychological and institutional resources to sustain care without compromising professionals’ mental health.^9^

Understanding the complexity of space, environment, interpersonal relations, and the health professional’s experience in the face of oncologic illness is essential for designing more humanized settings. In this horizon, categories such as “life,” “work,” “humanization,” “subjectivity,” “mental health,” and “stress/distress” have been examined by the Grupo de Pesquisa Vida e Trabalho, institutionalized in 1995 within the Conselho Nacional de Desenvolvimento Científico e Tecnológico and based at the Laboratory for the Humanization of Health Care at Universidade Estadual do Ceará.

This opinion article thus offers a critical reflection on the contributions of environmental psychology to creating restorative environments that promote humanized work among health professionals who care for people with cancer.

## METHODS

This opinion article adopts a qualitative, exploratory approach grounded in critical analysis of the scientific literature on how environmental psychology informs the creation of restorative environments and their impacts on humanized work among health professionals in oncology services. The methodological choice aims to elucidate interrelations between physical and psychosocial environments and workers’ mental health.^8^

To build the theoretical corpus, we conducted a bibliographic search across scientific articles, books, dissertations, and theses, focusing on contributions of environmental psychology to the design of restorative spaces and the humanization of care, with attention to the mental health of oncology professionals. Selection criteria emphasized thematic relevance and each source’s contribution to understanding the care environment as a determinant of well-being and stress mitigation in oncology settings.

Descriptors were defined using the Health Sciences Descriptors and included “environmental psychology,” “restorative environments,” “humanization of care,” “mental health,” “oncology,” and “health professionals.” No restrictions were applied regarding timeframe or language to ensure breadth and diversity of the analysis.

Throughout the process, we identified and selected key authors and studies examining links among the care environment, environmental psychology, mental health, and the humanization of work in oncology. This selection supported discussion and reflection on the care environment as an essential element in reducing stress and physical/mental fatigue among health professionals, thereby contributing to more compassionate and effective care for people with cancer.

The analysis was guided by two theoretical frameworks. First, the ART posits that environments — especially natural ones — help restore attentional capacity and reduce mental fatigue through soft fascination and distance from everyday pressures.^3^ Second, the SRT emphasizes that exposure to such environments elicits positive emotional responses, promoting stress relief, physical well-being, and emotional balance.^4^ Together, these frameworks inform how an appropriate care environment can mediate health workers’ engagement with the emotional demands of oncology care. Accordingly, the adopted methodology enabled not only a comprehensive literature search but also a critical, reflective analysis of ways to reframe the care environment in oncology services to strengthen humanized work and promote well-being and mental health among professionals involved in patient care.

## RESULTS AND DISCUSSION

The humanization of health care environments has been debated for decades, and the discussion has intensified as working conditions have become increasingly precarious, with workloads that hinder workers’ recovery and impede restorative processes in day-to-day care.^10^-^13^ In this section, we discuss the two principal theories in environmental psychology — ART and SRT — and examine how creating restorative environments can strengthen humanized work in oncology by promoting mental health, supporting professionals’ well-being, and improving the quality of care.^3^

## ART

ART posits that environments with nonthreatening stimuli — such as natural elements, diffuse lighting, and green landscapes — facilitate attention restoration. These features help recover attentional capacity after periods of cognitive overload during oncology workflows.^3^ Formulated in 1989 by Rachel and Stephen Kaplan and further developed in 1995 by S. Kaplan, ART directly engages with William James’s concepts of voluntary and involuntary attention and with landscape architect Frederick Law Olmsted’s propositions on the restorative benefits of natural environments.^3^,^9^^14^

Within oncology services, this principle can guide the design of waiting rooms as restorative spaces. Incorporating hanging vegetation, waterscape-inspired landscaping, nature-themed visual panels, and large windows overlooking green areas represents an architectural and symbolic care-environment strategy that can foster psychological restoration and enhance interprofessional relations among staff.^15^ Such resources support both patients and professionals, offering relief in the face of the high emotional and cognitive demands imposed by oncology treatment protocols.

## SRT

Certain environmental features support the restoration of psychological resources compromised under stress. Although stress is an adaptive response that prepares individuals to face threats, its persistence at high levels becomes dysfunctional, generating psychological distress and avoidance strategies.^4^ In this context, Ulrich argues that humans possess a biological predisposition to respond quickly and positively to restorative environmental contexts, which play a crucial role in psychophysiological recovery and in promoting health.^4^

SRT rests on four principles: i) being away, understood as the possibility of distancing oneself from everyday demands, thereby fostering attentional rest; ii) fascination, in which environmental stimuli capture attention spontaneously and effortlessly; iii) extent, the perception of a broad, coherent setting that invites exploration; and iv) compatibility, that is, alignment between environmental characteristics and individual needs, facilitating positive interactions.^9^

In oncology services, these principles can guide interventions such as i) creating quiet staff rest areas; ii) introducing circulation paths with natural light; iii) judicious use of color palettes at strategic points; iv) providing natural views in inpatient wards; v) establishing outdoor social spaces; and vi) implementing acoustic control (e.g., soft background music in chemotherapy units), all of which help to relieve day-to-day tension. In addition, offering integrative and complementary practices during breaks (e.g., auriculotherapy, aromatherapy, meditation, and massage) can potentiate the restorative effects of the care environment, promoting worker well-being and enhancing patients’ care experience.^16^

## FOUNDATIONAL REFLECTIONS

This interface invites reflection on the importance of valuing professionals within spaces that accommodate diverse demands and ensure comfort, functionality, and safety for all involved — dimensions closely linked to the brain’s influence on, and susceptibility to, the built environment.^17^ Across health services — especially in oncology (e.g., chemotherapy and radiotherapy units, inpatient wards, and palliative care) — the relevance of humanized practice and the care environment stands out, reconnecting physical and psychological settings and fostering autonomy and empowerment among workers and service users.^18^

Because of the complexity and emotional challenges that characterize oncology work, environmental psychology offers an essential strategy for guiding proposals to improve the work environment and promote the well-being of all involved. This includes optimizing spatial layout with functional floor plans, appropriate lighting, and acoustic control as well as incorporating natural elements — such as plants and green areas — to reduce stress/distress. Mental-health promotion can be reinforced by relaxation spaces and well-being programs; additionally, schedule flexibility and greater autonomy tend to increase job satisfaction.^19^,^20^

When discussing environmental influences on human behavior, it is important to recognize that environment is not limited to the material context in which actions occur; it comprises a set of stimuli that affect those actions consciously or unconsciously. People often do not perceive that they are being influenced by this subjective dimension of the environment.^21^

The places inhabited by professionals have been shaped by their own prior experiences and those of their predecessors. This historicity consolidates socially learned and transmitted spatial rules, from which diverse meanings emerge over time. There is interdependence among worker, environment, and temporality, with effects on behavior.^22^ This dynamic unfolds in worker–worker and worker–environment exchanges that are inseparable from the social context.^22^

**Figure 1:**
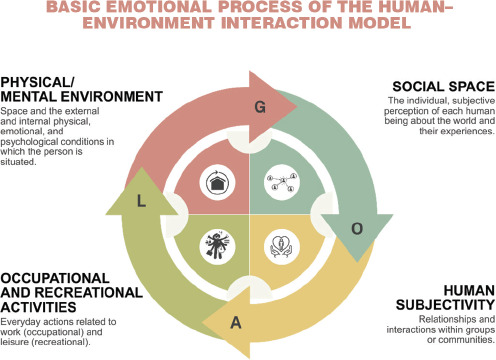
Human–Environment Interaction model. Source: adapted from Küller (1991).^23^

From this perspective, the Human–Environment Interaction model can be applied to oncology to understand how health professionals perceive the hospital care environment and the care experience.^23^ Professionals’ evaluations depend on their perception of the physical setting (infrastructure, comfort), interactions with the team (social space), and a sense of safety within the care process. Moreover, each patient is singular, with distinct needs and resources that shape the oncology-care experience (Figure 1).

Stress-inducing environments — marked by absent or inadequate environmental features and by precarious working conditions — tend to amplify the effects of chronic stress. Deficient lighting and ventilation, inadequate equipment, and the lack of rest areas generate discomfort and increase stress load.^24^

In parallel, labor precarization — manifested in job instability, weak legal protection of rights, insufficient pay, task overload, and limited organizational support — intensifies insecurity and psychological strain. Together, these factors create a setting conducive to chronic stress, heightening the risk of disorders such as anxiety and depression and underscoring the need for integrated interventions that combine environmental and organizational improvements to promote health and well-being.^25^,^26^

Attention restoration aligns with Brazil’s National Humanization Policy (PNH) by fostering work and care settings that integrate natural elements and restorative spaces — such as green areas and dedicated rest rooms.^27^ By prioritizing well-being and quality of work life, the PNH supports the creation of spaces that enhance compatibility, satisfaction, and safety while reducing stressors, thereby contributing to mental health and professional effectiveness. This articulation between attention restoration and humanization strengthens the development of welcoming, sustainable care environments that are essential for healthier and more effective clinical and work practices.^27^,^28^

Within care environments that evoke positive emotions and promote well-being, environmental psychology highlights the concept of restorative environments, emphasizing perception and subjectivity: “restorative” and/or “favorite” places are chosen based on lived experience, shaped by cultural and social components.^28^

The interrelations between oncology professionals and their surroundings reinforce the importance of designing spaces that promote mental health and well-being in a context of high emotional demand. While ART indicates that natural elements help reduce mental fatigue, insufficient infrastructure and labor precarization often exacerbate psychological distress among these professionals.^29^

## CONCLUSIONS

Humanizing oncology workspaces is essential to promoting the physical and psychological well-being of health professionals. Prioritizing a restorative care environment and creating spaces that ensure comfort, safety, and psychosocial support are fundamental to mitigating the impacts of treatment that is often prolonged, demanding, and stigmatized.

Human–environment interaction should guide the design of settings that provide not only physical support but also welcome and psychological care. Further studies are needed to investigate relationships among workers, the care environment, and mental health as well as the role of restorative environments, with the aim of advancing more effective and compassionate oncology care.

These investigations should interrogate institutional and organizational barriers to implementing restorative care environments and, specifically, explore stress indicators — such as cortisol levels and burnout indices — while assessing the effects of environmental interventions, including the incorporation of green areas in hospitals. Critical analyses can underpin interventions that move beyond stopgap solutions and consolidate effective practices responsive to the needs of both workers and patients.

In this horizon, environmental psychology is not only a theoretical framework but also a transformative instrument for informing institutional policies and hospital management strategies. Its recommendations can drive the creation of healthy, restorative environments aligned with the psychosocial needs of workers and patients, thereby strengthening the humanization and effectiveness of oncology care.
